# Advances and challenges of targeting epicardial adipose tissue (EAT) and perivascular adipose tissue (PVAT)

**DOI:** 10.1186/s12933-025-02763-z

**Published:** 2025-08-04

**Authors:** Dan Liu, Wujun Chen, Zhu Guo, Qun Gao, Bin Wang, Jie Wang, Weichao Hu, Chao Wang, Shuai Wang, Xiaolin Wu, Mantao Xu, Ganqiu Lan

**Affiliations:** 1 Guangdong Provincial People’s Hospital, Zhuhai Hospital (Jinwan Central Hospital of Zhuhai), Zhuhai, 519040 Guangdong China; 2 The Affiliated Hospital of Qingdao University, Qingdao Cancer Institute, Qingdao University, Qingdao, 266071 Shandong China; 3 Department of Endocrinology, Qilu Hospital (Qingdao), Cheeloo College of Medicine, Shandong University, Qingdao, 266000 Shandong China; 4 Department of Radiotherapy, School of Medical Imaging, Affiliated Hospital of Shandong Second Medical University, Shandong Second Medical Medical University, Weifang, 261053 Shandong China; 5 Department of Orthopedics, The Affiliated Hospital of Qingdao University (Pingdu), Qingdao, 266000 Shandong China; 6 College of Information Technology, Shanghai Jian Qiao University Co Ltd, No.1111 Hucheng Ring Rd, China (Shanghai) Pilot Free Trade Zone Lin-Gang Special Area, Shanghai, 201315 China

**Keywords:** Atherosclerosis, EAT, PVAT, Adipose tissue delivery, saRNA, Agent development

## Abstract

Clinical consensus statements have been issued for various adipose tissues (ATs), particularly regarding the diagnostic value of epicardial AT (EAT) and perivascular AT (PVAT) in cardiovascular disease. PVAT and EAT are promising targets for drug development and many drugs were also investigated, including DPP4 inhibitors, GLP-1R agonists, and SGLT-2i. Notably, DPP4 was the only gene that highly expressed in EAT and PVAT. DPP4 also reduced GLP-1 and its receptor GLP-1R expression, suggesting that DDP4 is a promising target for targeting EAT and PVAT. However, these preparations have poor specificity for PVAT and EAT. AT delivery strategies or specific AT genes, such as ADIPOQ and PHB1, may solve these problems. ADIPOQ is only expressed in AT and encodes adiponectin (ADPN). PHB1 is an AT vascular biomarker. Many ADPN and PHB1 agents have also been developed in preclinical and clinical trials. However, these agents have serious off-target effects. SaRNA, an RNA activation technology, may reduce off-target effects. Several saRNA agents were also developed in preclinical and clinical trials. Direct overexpression of ADIPOQ and PHB1 through saRNA in combination with extrahepatic delivery materials may be beneficial for drug development. This review focuses on recent advances targeting EAT and PVAT agents and identifies new therapeutic targets.

**Graphical abstract:**

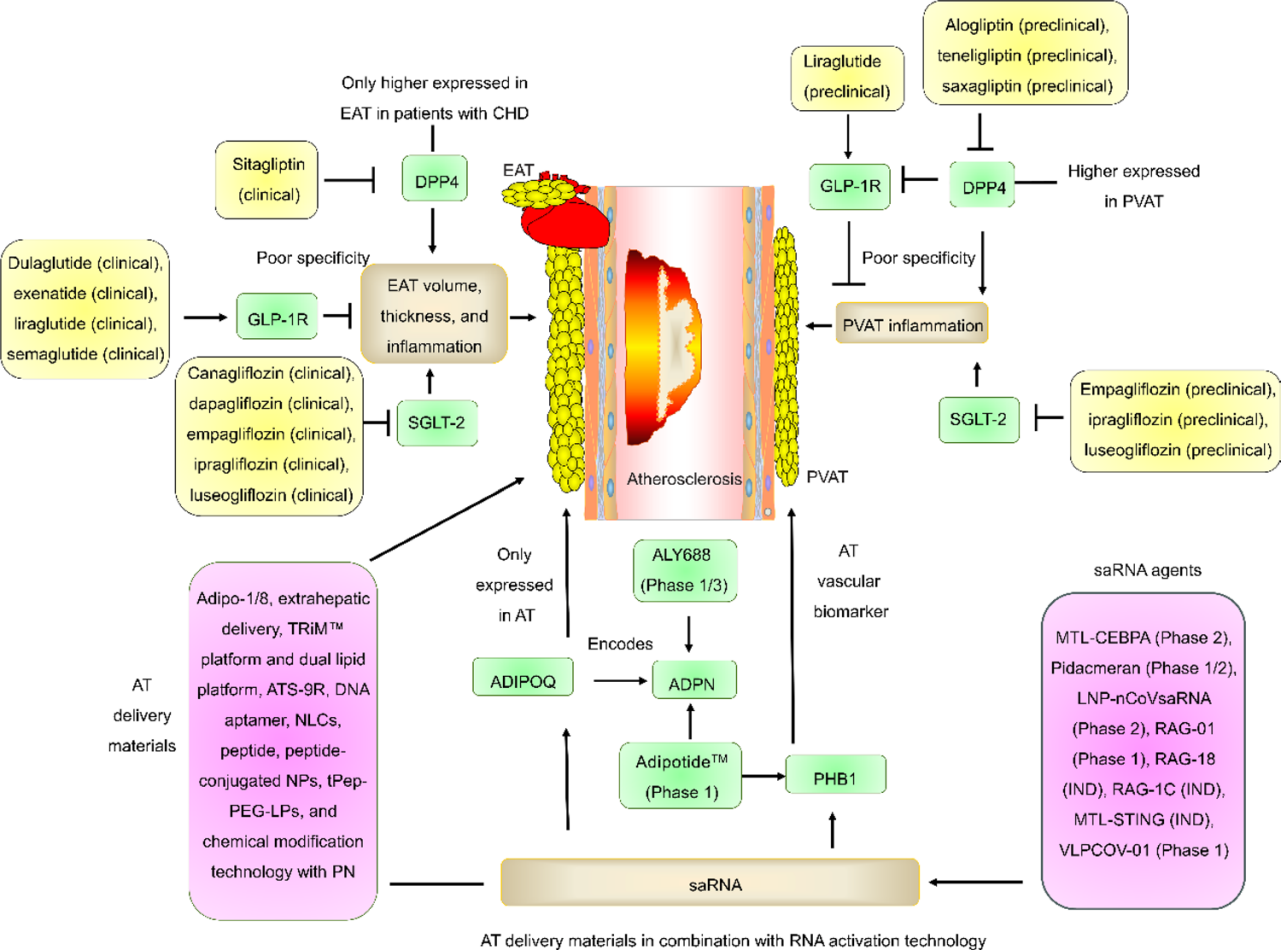

## Introduction

Aging is a common risk factor for the occurrence and development of many diseases, such as coronary heart disease (CHD), and a key problem that threatens the health and life expectancy of the elderly population [1–3]. The lifespan of humans has been greatly extended by improvements in social and economic conditions. By 2050, the worldwide population aged 60 years and older is expected to total 2 billion, up from 900 million in 2015 according to the WHO. Atherosclerosis is the main cause of CHD and is characterized by the formation of lipid-laden plaques in large- and medium-sized vessels [4–8]. Age at the time of death was shown to be positively correlated with atherosclerosis, and only aging was associated with an increased risk of severe atherosclerosis [9]. Age-related atherosclerosis is the presumed cause of 40% of all deaths and the leading cause of death in elderly populations [10, 11]. Patients who are actively treated with statins still have residual cardiovascular risk even when low-density lipoprotein cholesterol (LDL-c) is met or even drops below 70 mg/dL [12]. Therefore, identifying unknown therapeutic targets and developing new antiatherosclerotic agents are challenging.

**Fig. 1:**
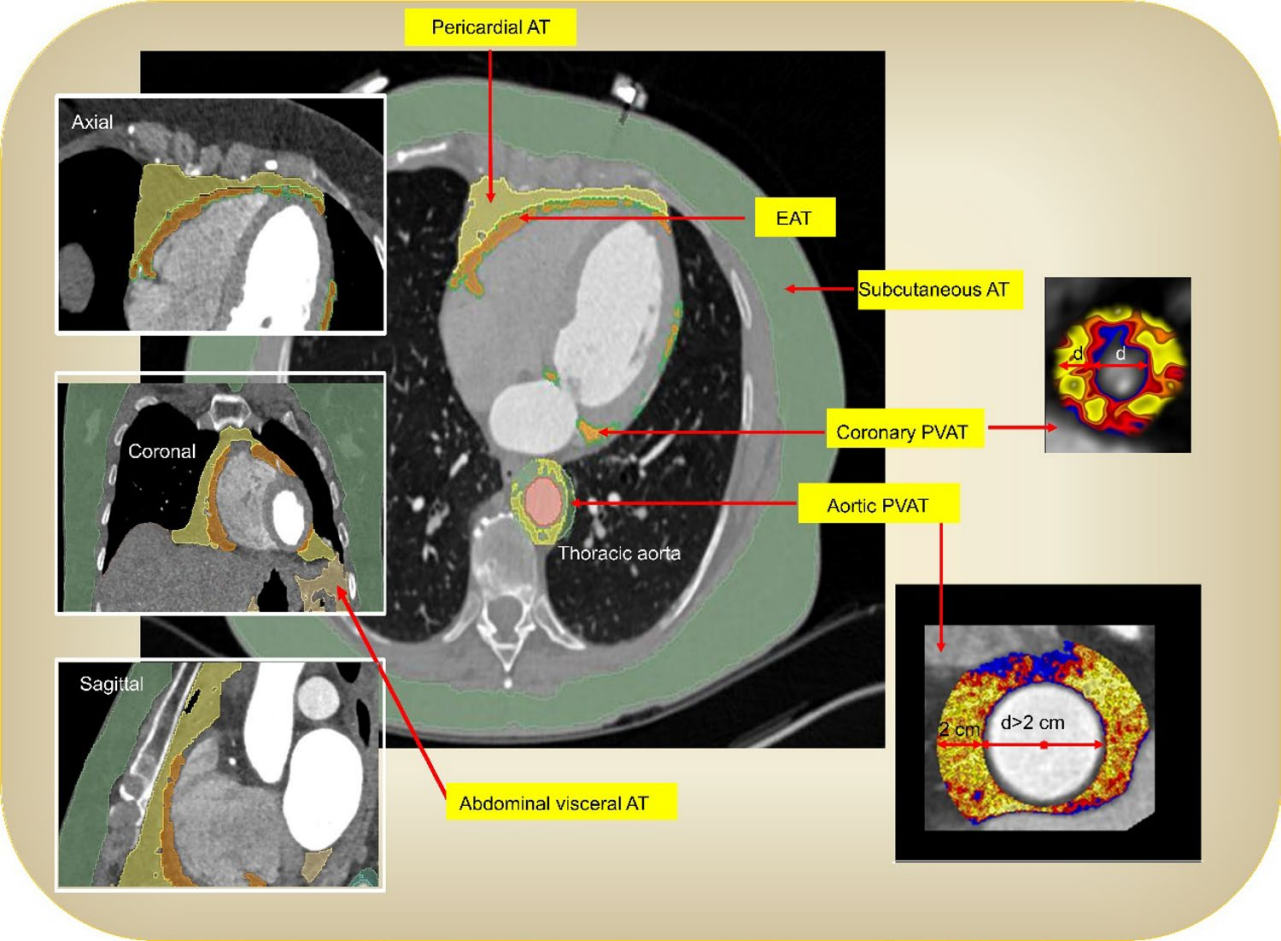
Imaging of human AT depots by CT. This information was modified from Ref [22]

The pathological characteristics of aging-related atherosclerosis include vascular aging, lipid accumulation, and inflammation. Controlling lipids and inflammation is essential for preventing atherosclerosis [13–15]. Interestingly, adipose tissue (AT) is stored not only in the subcutaneous abdomen but also in nonadipose organs, including muscle, liver, pancreas, heart, and blood [16–19]. Normal aging is associated with a gradual increase in AT, which usually peaks in men at approximately age 65 and later in women. AT contains many different cell and tissue types, including macrophages, epicardial AT (EAT), perivascular AT (PVAT), and visceral AT (Fig. 1 and Table 1) [20, 21]. Clinical consensus statements have been issued for various ATs, particularly regarding the diagnostic value of EAT and PVAT in cardiovascular disease [22]. Indeed, EAT and PVAT play a key role in aging-related atherosclerosis development [23, 24]. Many drugs, such as dipeptidyl peptidase 4 (DPP4) inhibitors, glucagon-like peptide-1 (GLP-1) receptor (GLP-1R) agonists, and sodium glucose cotransporter 2 (SGLT-2) inhibitors (SGLT-2i), also inhibit atherosclerosis development by reducing the proinflammatory response in EAT and PVAT. However, these preparations have poor specificity for PVAT. In this review, we discuss the biological roles of EAT and PVAT in vascular disease, and we focus on their potential as targets for therapeutic intervention.

**Table 1:** The tissue types of AT. This information was modified from Ref [22]

Name	Definition
EAT	The AT between the visceral pericardium and the myocardium and includes the PVAT around the coronary arteries
Paracardial AT	AT is located in near the heart
Pericardial AT	The chest’s AT that surrounds the heart is located outside the pericardial sac (excluding periaortic AT)
PVAT	AT is located in around blood vessels with a radial distance of less than or equal to 2 cm from the vessel wall
Subcutaneous AT	AT is located in subcutaneous (skin), including the subcutaneous chest, abdomen, buttocks, and femur
Thoracic AT	AT is located in the pericardium, nonpericardium chest, epicardium, and around blood vessels (around any blood vessel, such as coronary arteries, aorta, and internal mammary arteries, etc.)

## The role of EAT and PVAT in aging-related atherosclerosis

### EAT

Heart AT (HAT) is AT in the heart and around the heart, namely intra-myocardial AT (IMAT) and EAT, respectively. IMAT is associated with left ventricular thickness and heart failure. The EAT is positively correlated with atherosclerosis and myocardial infarction (MI) [25–27]. The EAT is the AT surrounding the arcus aorta, coronary arteries, ventricles, and apex of the heart. The EAT shares a common blood supply with the myocardium and affects cardiac function. EAT thickness is clinically associated with atherosclerotic cardiovascular disease (ASCVD) factors, such as plasma cholesterol, the intima-media thickness of carotid artery (measured by carotid ultrasonography manually or automatically), and coronary calcification [28, 29]. EATs promote atherosclerosis development by releasing proinflammatory and proatherogenic factors, including angiotensinogen (AGT), IL-6, leptin, monocyte chemoattractant protein-1 (MCP-1, also named CCL2), nerve growth factor (NGF), resistin, omentin, plasminogen activator inhibitor-1 (PAI-1, also known as serpin E1), TNFα, and visfatin. Notably, EAT is a source of NF-κB and JNK-mediated inflammation [30]. A phenotype conversion of EAT from BAT to WAT was found in the progression of atherosclerosis. IL-6 promoted the focal occurrence and development of atherosclerosis by inducing this conversion via activating JAK/STAT3 pathway [31]. There is no fibrous fascial layer to impede the diffusion of free fatty acids and adipokines between EAT, the underlying vessel wall, and the myocardium. These proatherogenic factors readily influence coronary atherogenesis and myocardial function. Thus, reducing EAT could help in preventing coronary artery disease (CAD). Notably, IMATs and EATs are independent of obesity. EAT thickness has been independently associated with atherosclerotic plaque burden and coronary calcification, suggesting that EAT is a potential risk factor for ASCVD. Age-related body fat mass, ectopic fat, heart AT (HAT), and PVAT were measured (Table 2) [22, 32]. The EAT may prove to be a more sensitive biomarker of ASCVD [33–35]. However, determining how to conduct noninvasive standard assessments is key.

**Table 2:** The morphology of chest AT and its association with aging and cardiovascular risk. This information was modified from Ref [22, 25–27]

Name	CT	Changes with aging	Cardiovascular risk
EAT		Increasing	High
Pericardial AT		Increasing	High
PVAT		Increasing	High
Subcutaneous AT		Decreasing	High

### PVAT

**Fig. 2:**
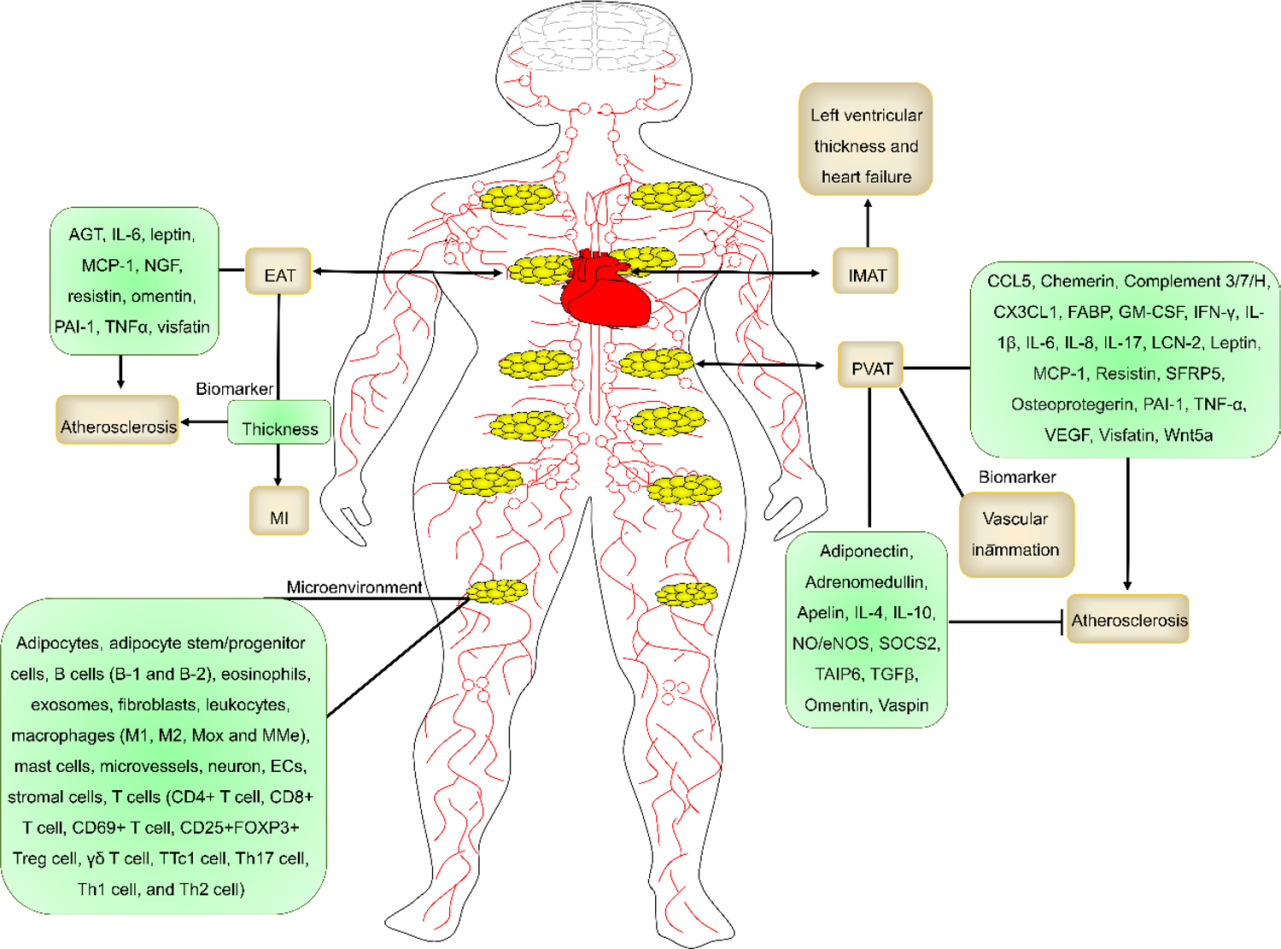
The role and factors of EAT and PVAT in atherosclerosis. The AT microenvironment includes multiple cell subtypes (e.g., adipocytes). EAT thickness is a potential biomarker of atherosclerosis and promotes atherosclerosis development by releasing proinflammatory factors (e.g., TNFα). IMAT thickness is a potential biomarker of left ventricular thickness and heart failure. PVAT is a potential biomarker of vascular inflammation and promotes or suppresses atherosclerosis development by releasing/expressing proatherogenic factors (e.g., IL-1β) or anti-atherogenic factors (e.g., ADPN)

PVAT is the AT surrounding different blood vessels in addition to capillaries, pulmonary vessels, and cerebrovascular vessels. Blood vessels are divided into three layers: the intima, media, and adventitia. The adventitia is also divided into two sublayers: the adventitial compacta and the PVAT (adventitial fat) [36]. In fact, there are no obvious anatomical boundaries between coronary PVAT and EAT in humans. PVAT deposition is strongly related to worse health conditions in elderly people. PVAT regulates atherosclerosis development by clearing FFAs and releasing paracrine factors associated with vascular tone, inflammation, redox state, and VSMC proliferation [37]. With aging, increased inflammation in the PVAT is observed and contributes to inflammation and atherosclerosis. PVAT is an important source of oxidative stress and inflammation in individuals with obesity, which may result in vascular dysfunction. Activating autophagy in PVAT helps to regulate the function of PVAT and thereby improve vascular function in individuals with obesity [38, 39]. PVAT can release several proatherogenic mediators, including CCL5 (RANTES), chemerin, complement 3/7/H, CX3CL1, FABP, GM-CSF, IFN-γ, IL-1β, IL-6, IL-8, IL-17, LCN-2, leptin, MCP-1, resistin, osteoprotegerin, PAI-1, TNFα, VEGF, visfatin, and Wnt5a. PVAT can also release several antiatherogenic mediators, including ADPN, adrenomedullin, apelin, IL-4, IL-10, NO/endothelial NO synthase (eNOS), SFRP5, SOCS2, omentin, TAIP6, TGFβ, and vaspin (Fig. 2) [22, 40]. However, these factors generally have systemic effects and may play different roles depending on the disease state. How to target factors in PVAT is the key. Notably, NLRP3 inflammasome promoted vascular aging by inducing PVAT dysfunction [41]. GLP-1 reduced NLRP3 inflammasome-dependent inflammation by inhibiting the NF-κB pathway in PVAT [42]. Therefore, the key inflammatory pathways, such as NF-κB, JAK/STAT, and inflammasome activation, play a key role in EAT and PVAT-mediated vascular inflammation.

## Clinical or preclinical drugs targeting EAT and PVAT for the treatment of aging-related atherosclerosis

### Targeting EAT

#### DDP4 inhibitors

**Fig. 3:**
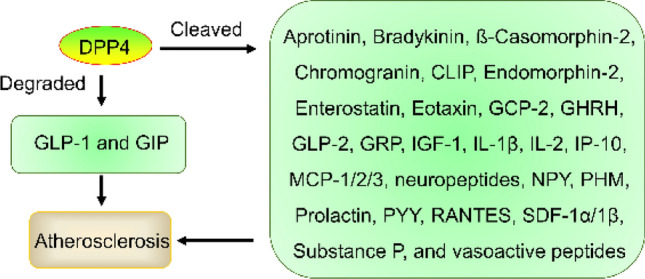
The role and mechanism of DPP4 in atherosclerosis. DPP4 promotes atherosclerosis development by degrading GLP-1 and GIP and cleaving aprotinin, bradykinin, CLIP, CCL11, GCP-2, IL-2, and IL-1β

DPP4 is a ubiquitous multifunctional glycoproteic protease that regulates glucose and lipid metabolism, immunology, and nutrition [43]. DPP4 degrades GLP-1 and glucose insulinotropic peptide (GIP, also known as incretin) within a few minutes. Incretins stimulate insulin while inhibiting glucagon secretion. Thus, DDP4 inhibitors improved glycemic control by preventing GLP-1 inactivation to increase GLP-1 concentrations in type 2 diabetes mellitus (T2DM) patients [44]. In addition, DPP4 cleaves chemokines and cytokines (Fig. 3), including stromal-derived factor (SDF)-1α/1β, neuropeptides, vasoactive peptides, aprotinin, bradykinin, ß-casomorphin-2, chromogranin, CLIP, endomorphin-2, enterostatin, eotaxin (or CCL11), GCP-2 (or CXCL6), GHRH, GRP, IGF-1, prolactin, IL-2, IL-1β, IP-10, MCP-1/2/3, RANTES, NPY, substance P, PYY, PHM, and GLP-2 [45]. These factors play a key role in atherosclerosis. As mentioned above, GLP-1 increased ADPN expression and M2 macrophage polarization and reduced lipid accumulation in ATs. Thus, DPP4 inhibition has the potential to affect CVD.

##### Sitagliptin

Sitagliptin has been approved for the treatment of adult patients with T2DM in more than 130 countries worldwide. Human and animal experiments have shown that sitagliptin also suppressed atherosclerosis development [46, 47]. Sitagliptin also regulated EAT function. Sitagliptin reduced EAT thickness by 15% in overweight/obese patients with T2DM who were inadequately controlled on metformin monotherapy [48]. Sitagliptin reduced the pericardial, epicardial, and paracardial fat content in 44 Japanese patients with early-stage T2DM without CVD complications [49]. Indeed, DPP4 was more highly expressed only in adipocytes isolated from EAT but not in adipocytes isolated from subcutaneous fat in 25 patients with CHD, suggesting that inhibiting EAT function is one of the major pharmacological mechanisms by which DPP4 inhibitors suppress atherosclerosis development. Notably, other DPP4 inhibitors, including alogliptin, dutogliptin, gemigliptin, linagliptin, omarigliptin, saxagliptin, teneligliptin, trelagliptin, and vildagliptin, have also been approved for the treatment of T2DM [50]. These inhibitors also reduced atherosclerosis development. However, the effects of these inhibitors on EAT function have not been investigated. Hence, further studies are needed to evaluate the efficacy of targeting DPP4 on EAT function.

#### GLP-1R agonists

GLP-1 is a hormone that promotes insulin and suppresses glucagon secretion in a glucose-dependent manner by binding to GLP-1R which is expressed in cardiac and vascular tissues [51, 52]. GLP-1 regulates heart and artery wall function by activating a variety of signaling pathways via binding to GLP-1R, including the PI-3 K/Akt, cAMP/ERK, MAPK, eNOS, and cAMP/PKA/CREB pathways. Interestingly, EAT and adipocytes also express GLP-1 and GLP-1R. GLP-1 could increase the expression of the vasoprotective hormone ADPN and M2 macrophage polarization and reduce lipid accumulation in AT, suggesting that the GLP-1/GLP-1R axis plays a key role in protection against cardiovascular disease [53–55]. GLP-1R agonists are a new class of antidiabetic drugs used to treat T2DM. Importantly, GLP-1R agonists, such as liraglutide, semaglutide, exenatide, dulaglutide, and sitagliptin, suppress coronary atherosclerosis by reducing EAT volume and ameliorating the inflammatory phenotype of EAT [56, 57].

##### Liraglutide

Liraglutide was approved for use in patients ≥ 10 years old with T2DM by the FDA [58]. Liraglutide was also approved by the FDA to reduce the risk of major adverse cardiovascular events in adults with T2DM. Liraglutide reduced EAT thickness with raging from 29 to 36% in patients with T2DM [59, 60]. Liraglutide also reduces cardiac AT in patients with T2DM [61]. However, some colleagues have questioned these findings and believe that weight loss interventions, including EAT, are expected to preferentially mobilize visceral fat [62]. Other studies have shown that liraglutide did not change EAT thickness in patients with T2DM [63, 64]. Notably, this view is questionable or at least controversial. Subcutaneous fat loss is always greater than visceral fat loss, regardless of weight loss interventions [65]. In addition, other visceral fat markers were not measured, and the dosage, duration of treatment and patients also varied [59]. Liraglutide causes a massive and rapid reduction in cardiac fat independent of weight loss in individuals with T2DM [66]. Therefore, liraglutide could increase epicardial fat lipolysis and reduce fat mass [65].

##### Semaglutide, exenatide, and dulaglutide

Semaglutide, exenatide, and dulaglutide were approved for the treatment of T2DM. Semaglutide was also approved for reducing the risk of major adverse cardiovascular events (such as cardiovascular death, nonfatal MI, and nonfatal stroke) in patients with T2DM and established cardiovascular disease by the FDA in January 2020. Semaglutide, exenatide, or dulaglutide can induce rapid, substantial, and dose-dependent reductions in EAT thickness in patients with T2DM and obesity [60, 67, 68]. Semaglutide and exenatide reduced atherosclerosis development in clinical and preclinical studies [69, 70], suggesting that inhibiting EAT function is one of the pharmacological mechanisms by semaglutide and exenatide suppress atherosclerosis development. However, other studies have shown that exenatide did not modify carotid plaque volume or composition [71]. More studies are needed to confirm the effect of exenatide on plaque in patients.

Dulaglutide was effective for both primary and secondary cardiovascular prevention in patients with T2DM and CVD. However, dulaglutide failed to reduce the aortic plaque area in non-diabetic mice, suggesting that dulaglutide has cardiovascular protective effects on T2DM patients with ASCVD. Notably, tirzepatide, the dual GLP-1R/GIPR agonist, could improve cardiovascular risk biomarkers and diabetes-related cardiac damage [72, 73]. Tirzepatide reduced pericardial AT volumes in patients with heart failure with preserved ejection fraction (HFpEF) and obesity. However, tirzepatide did not change EAT volumes [73]. Other GLP1-R agonists, including lixisenatide and albiglutide, also reduced atherosclerosis development. However, the effect of these GLP1-R agonists on EAT function has not been investigated. More studies are needed to evaluate the effect of these GLP1-R agonists on EAT function.

#### SGLT-2 inhibitors

SGLT-2, a member of the sodium-glucose cotransporter family, is encoded by SLC5A2. SGLT-2 transports glucose from the renal tubule lumen to renal tubule epithelial cells. SGLT-2i are a newly identified class of drugs for the treatment of T2DM [74, 75]. The major pharmacological action of SGLT-2i involves inhibiting glucose reabsorption in the kidney and promoting glucose excretion by targeting SGLT2. In addition to its direct pharmacological action, SGLT-2i, including canagliflozin, dapagliflozin, empagliflozin, ipragliflozin, and luseogliflozin, also suppressed the coronary atherosclerosis process [56, 57].

##### Canagliflozin

Canagliflozin was approved by the FDA to reduce the risk of end-stage kidney disease (ESKD), doubling of serum creatinine, cardiovascular death, and hospitalization for HF in patients with T2DM and diabetic nephropathy with albuminuria [76]. Canagliflozin could reduce the risk of major adverse cardiovascular events in patients with T2DM combined with ASCVD (NCT03962686) [77]. Canagliflozin reduced the thickness of EAT in patients with T2DM independent of its effect on reducing blood glucose [78], suggesting that canagliflozin reduces EAT-mediated atherosclerosis progression independent of reducing T2DM treatment.

##### Dapagliflozin

Dapagliflozin was approved for the treatment of T1DM and T2DM [79, 80]. Dapagliflozin also reduced the rate of cardiovascular death or hospitalization for heart failure and ASCVD [81]. Dapagliflozin reduced the EAT volume/thickness and TNFα level in T2DM patients with ASCVD [82–84]. Changes in EAT thickness and body weight in these patients were not significantly correlated, suggesting that dapagliflozin reduced EAT reduction thickness independent of weight loss [83]. Dapagliflozin increased glucose uptake by increasing glucose transporter GLUT-4 in the EAT of patients who underwent cardiac surgery [85]. Dapagliflozin also reduced the secretion of proinflammatory chemokines, including CXCL8, CCL5, and MCP-1, which promote atherosclerosis development in patients with EAT. Dapagliflozin improved the differentiation of stromal vascular cells (SVCs) in these patients’ EATs [86], suggesting that one of the major pharmacological mechanisms of dapagliflozin is through the inhibition of EAT function.

##### Empagliflozin

Empagliflozin was approved by the FDA for the treatment of T2DM and reduced the risk of cardiovascular death in adults with T2DM and established CVD. Empagliflozin reduced the EAT volume and aortic stiffness in nondiabetic patients with heart failure with reduced ejection fraction (HFrEF) [87, 88]. Empagliflozin in combination with sitagliptin changed the fat content of pericardial, epicardial, and paracardial in patients with early-stage T2DM without CVD complications [49, 89]. Sitagliptin reduced the EAT volume in overweight/obese patients with T2DM, suggesting that empagliflozin reduced the EAT volume. However, some studies refuted the cardiovascular protection of empagliflozin. For example, empagliflozin reduced liver AT by 55% but did not affect EAT in high-fat/high-sucrose (HFHS) diet-fed mice. Empagliflozin significantly reduced liver AT and visceral fat by 92.6% and 98.7%, respectively, but did not affect EAT in patients with T2DM [90]. Further studies are needed to confirm the role of empagliflozin in EAT.

##### Ipragliflozin

Ipragliflozin was approved for the treatment of T2DM in Japan [91, 92]. Ipragliflozin reduced EAT accumulation in patients with visceral obesity and nonobese with T2DM [93]. Ipragliflozin also reduced the carotid intima-media thickness, which is a surrogate marker of carotid atherosclerosis, in patients with T2DM [94]. Therefore, inhibition of EAT function is a major pharmacological mechanism of ipragliflozin.

##### Luseogliflozin

Luseogliflozin was approved for the treatment of T2DM in Japan [95]. Luseogliflozin reduced EAT volume and CRP-mediated systemic microinflammation but not visceral fat area in Japanese patients with T2DM [57], suggesting that canagliflozin reduces atherosclerotic progression in diabetic patients by reducing EAT-mediated proinflammatory processes.

Taken together, SGLT-2i have cardiovascular protective effects on diabetes patients diagnosed with a greater risk of atherosclerotic cardiovascular disease. One of the major pharmacological mechanisms of SGLT-2i is through the inhibition of EAT function. However, the effect of SGLT-2i on reducing atherosclerosis was independent of SGLT-2 reduction. The role of SGLT-2 in atherosclerosis has not been investigated. Further studies, such as knockout and siRNA, are needed to confirm the effect of SGLT-2 on atherosclerosis. In addition, other SGLT-2i, including ertugliflozin, remogliflozin, sergliflozin, sotagliflozin, and tofogliflozin, also reduced the accelerated atherosclerosis associated with diabetes. However, the effect of these SGLT-2i on EAT function has not been investigated. Further studies are needed to confirm the effect of these SGLT-2i on EAT function.

### Targeting PVAT

#### DPP4 inhibitors

DPP4 was expressed in PVAT. PVAT secretes DPP4, which originates from adipocytes and then promotes atherosclerosis development by enhancing proinflammatory factors, including IL-6, TNFα, and IL-1β [42, 96, 97]. DPP4 also promotes atherosclerosis development by suppressing GLP-1, which is involved in crosstalk loops between PVAT and atherosclerosis [98, 99]. DPP4 inhibitors, such as alogliptin, saxagliptin, and teneligliptin, have displayed a range of benefits for the treatment of ASCVD by targeting PVAT. Thus, targeting DPP4 is a crucial regulator of the crosstalk between PVAT and atherosclerosis.

##### Alogliptin

Alogliptin was approved for the treatment of T2DM [100]. Alogliptin enhanced autophagy in the PVAT by enhancing the GLP-1/GLP-1R/light chain 3B-II (LC3B-II, a biomarker of autophagy) axis in HFD-fed mice. The GLP-1R antagonist exendin abolished alogliptin-induced autophagy and vasodilation in PVAT [101], suggesting that GLP-1R is necessary for the beneficial effects of GLP-1 and alogliptin on PVAT. The activation of autophagy in PVAT by alogliptin leads to improved vasodilation via the Akt-eNOS signaling pathway, increasing ADPN levels, and decreasing TNFα levels in PVAT [101, 102]. Many studies have shown that PVAT is an important source of autophagy, vasodilation, and inflammation in individuals with obesity and atherosclerosis [103]. Autophagy suppresses the progression of atherosclerosis by regulating inflammatory responses and foam cell formation, suggesting that alogliptin attenuates inflammation and foam cell formation in PVAT. Vasodilation function also plays a key role in reducing atherosclerosis development [104, 105]. Thus, alogliptin suppressed the progression of atherosclerosis by inducing autophagy and vasodilation in PVAT.

Alogliptin also reduces plaque inflammation and monocyte activation/chemotaxis by reducing DDP4 expression [106]. As mentioned above, DPP4 is a crucial regulator of the crosstalk between PVAT and atherosclerosis by suppressing GLP-1. Based on the role of alogliptin in enhancing GLP-1 in PVAT, we hypothesize that alogliptin increased GLP-1 expression by suppressing DPP4 in PVAT. However, the effect of alogliptin on DPP4 in PVAT has not been investigated. Notably, alogliptin increased hospital admissions for HF by 19%, although the difference was not statistically significant. The potential increase in HF risk associated with alogliptin was released by the FDA for drug safety communication on 5 April 2016 [107]. Further studies are needed to confirm the safety of alogliptin in treating ASCVD in clinical trials.

##### Saxagliptin

Saxagliptin was approved for the treatment of T2DM [108]. Many studies have shown that arterial stiffness is a risk factor for aging and ASCVD [109], and detection biomarkers of arterial stiffness are useful tools for identifying early ASCVD, adverse clinical outcomes in young adults, and patients with a modest risk factor profile [110–112]. Coronary PVAT promotes aortic stiffness during aging and hypercholesterolemia by increasing TNFα and IL-10 levels in PVAT by enhancing collagen deposition within the artery [113–115]. Interestingly, saxagliptin reduced coronary vascular stiffness in aortic-banded mini pigs by inhibiting PVAT inflammation. Specifically, saxagliptin decreased the levels of advanced glycation end products (AGEs), nitrotyrosine, NF-κB and its downstream molecules TNFα and IL-10 in coronary PVAT, thereby reducing PVAT inflammation and coronary vascular stiffness [116]. However, saxagliptin had no effect on the risk of cardiovascular death, MI, or ischemic stroke in T2DM patients with an HbA1c of 6.5%-12.0% (SAVOR-TIMI 53 Trial) [117]. The rate of hospitalization for heart failure with saxagliptin treatment was significantly increased by 27% [118]. For safety reasons, FDA drug safety communication revealed a potential increase in HF risk with saxagliptin treatment on 5 April 2016 [107]. Further studies are needed to confirm the efficacy and safety of saxagliptin in treating ASCVD.

##### Teneligliptin

The use of teneligliptin for the treatment of T2DM is already on the market in Japan and Korea [119]. Teneligliptin also has shown cardiovascular protective effects. Teneligliptin suppresses atherosclerosis by reducing the proinflammatory response in PVAT. Specifically, teneligliptin suppressed the expression of inflammatory factors, including F4/80, VCAM-1, ICAM-1, and Nox4, in the PVAT around the aortic arch of apoE-/- mice [120]. Downregulation of the pro-inflammatory response in PVAT is one of the pharmacological mechanisms underlying the antiatherosclerotic effect of teneligliptin. However, the effect of teneligliptin on ASCVD in the clinic has not been investigated. Further clinical studies are needed to confirm the efficacy of teneligliptin for treating ASCVD.

#### SGLT-2i and GLP-1R agonists

SGLT-2i, such as empagliflozin [121], ipragliflozin [122], and luseogliflozin [123], inhibited atherosclerosis development by reducing the proinflammatory response in PVAT. The role of GLP-1/GLP-1R in PVAT has been reviewed [124]. However, only one GLP-1R agonist liraglutide was investigated in PVAT [125].

**Fig. 4:**
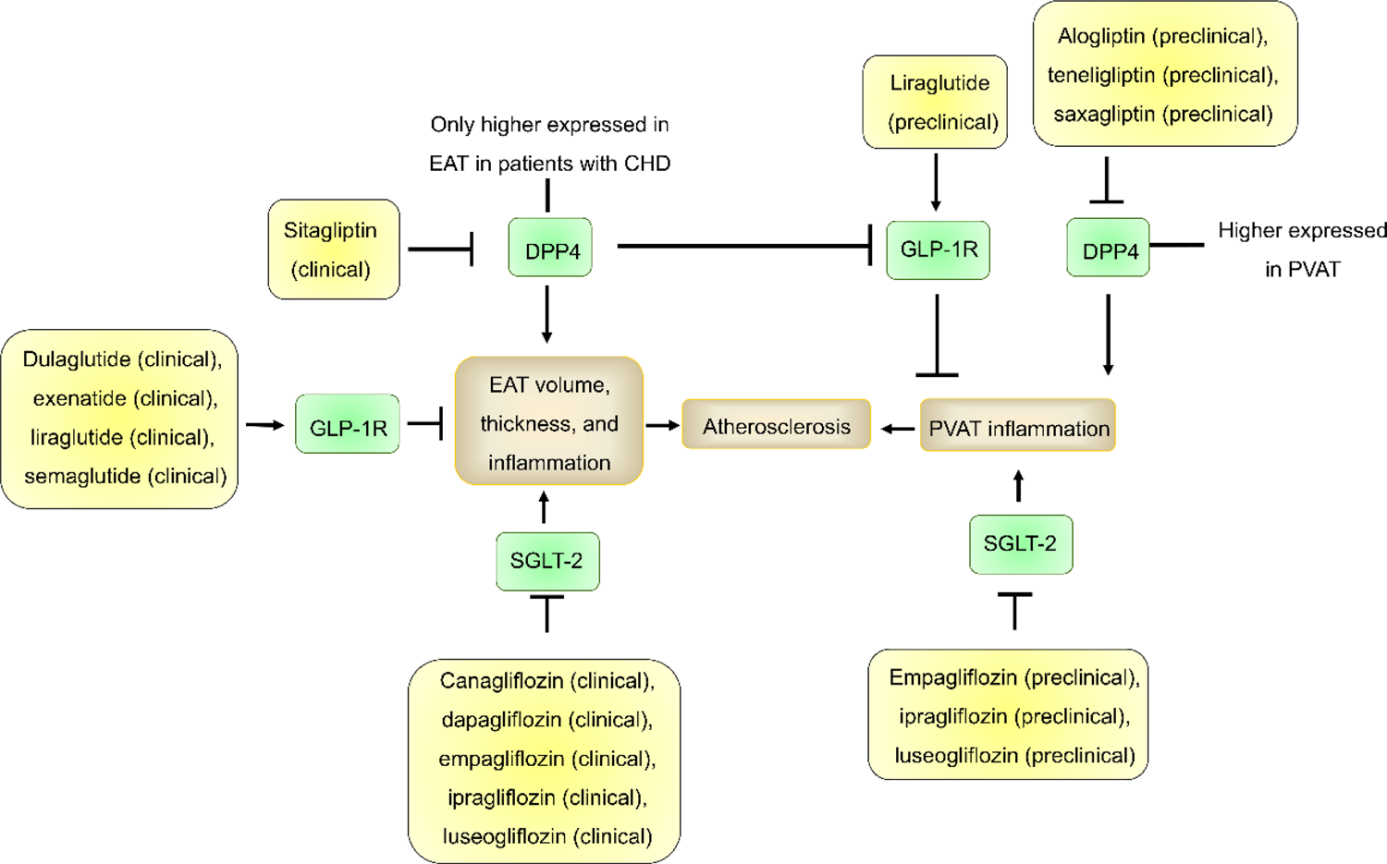
Clinical or preclinical drugs targeting EAT and PVAT for the treatment of atherosclerosis. Many drugs, such as DPP4 inhibitors, GLP-1R agonists, and SGLT-2i, inhibit the development of atherosclerosis by targeting EAT and PVAT. Only DPP4 was more highly expressed in EAT in patients with CHD and reduced GLP-1R expression

Taken together, many drugs, such as DPP4 inhibitors, GLP-1R agonists, and SGLT-2i, inhibit the development of atherosclerosis by targeting EAT and PVAT (Fig. 4), suggesting that EAT and PVAT are promising targets for drug development. However, these preparations have poor specificity for PVAT and EAT due to they have another mechanism for regulating atherosclerosis.

## Targeting AT delivery, specific EAT/PVAT targets, and RNA activation technology: important breakthroughs in drug development

### AT delivery strategies

Given that the available drugs have poor specificity for PVAT. We hypothesize that the development of specific PVAT preparations is an important breakthrough direction in the future. At present, two strategies may be able to solve the problem of specific PVAT and EAT preparations. (1). Adopting targeted AT delivery strategies (Table 3). (2). Targeting specific AT genes, such as ADIPOQ and prohibitin 1 (PHB1). AT delivery strategies included, including Adipo-1 [126], Adipo-8 [126, 127], Alnylam's extrahepatic delivery (such as the INHBE gene, Centyrin ligands, 2′-O-hexadecyl (C16) conjugates, N-acetylgalactosamine (GalNAc) conjugates, GEMINI™ platform (Bis-RNAi™) [128–133], Arrowhead's Targeted RNAi Molecule (TRiM™) platform [134] and dual lipid platform [135], ATS-9R [136], DNA aptamer [137], nanostructured lipid carriers (NLCs) [138], peptide [137, 139, 140], peptide-conjugated NPs [141], PTNP [142], tPep-PEG-LPs [143], and Wave Life Sciences' chemical modification technology (such as GalNAc conjugates) with phosphoryl guanidine (PN) [144]. However, more studies are needed to confirm which technology/materials are more suitable for mass production, good safety, and clinical applications.

**Table 3:** The technique for AT delivery

Name	Sequence/introduction	Refs
Adipo-1	ATGAGAAGCGTCGGTGTGGTTACTCCGGGCACTTGATATATCGATATGGAGAATAATGCACCCTGAGCGGGCTGGCAAGGCGCATA, A aptamer using cell-SELEX technology (specifically bind to mature adipocytes, human lung adenocarcinoma epithelial A549 cell line, pancreatic cancer PL45 cell line, and breast cancer MCF-7 cell line)	[126]
Adipo-8	ATGAGAAGCGTCGGTGTGGTTAAACACGGAACGAAGGTGCAGGAAGATTTGTCGATGCGGTGCCTGAGCGGGCTGGCAAGGCGCATA, A aptamer using cell-SELEX technology (specifically bind to mature adipocytes and APMAP, a crucial factor in adipogenesis and mature adipocytes biomarker)	[126, 127]
Alnylam’s extrahepatic delivery	Centyrin ligands, 2′-O-hexadecyl (C16) conjugates, N-acetylgalactosamine (GalNAc) conjugates, GEMINI™ platform	[128–133]
Arrowhead’s extrahepatic delivery	TRiM™ platform, dual lipid platform	[134, 135]
ATS-9R	C-KGGRAKD-RRRRRRRRR-C (specifically bind to prohibitin, an AT vascular biomarker). This information was modified from Ref [136]	[136]
DNA aptamer	ATGAGAAGCGTCGGTGTGG	[137]
DNA aptamer	TTAAACACGGAACGAAGGT	[137]
DNA aptamer	GCAGGAAGATTTGTCGATG	[137]
NLCs	Size: 121 nm	[138]
Peptide	P3: CKGGRAKDC (specifically bind to prohibitin)	[137, 139, 140]
Peptide	CPATAERPC	[137]
Peptide	CGLHPAFQC	[137]
Peptide	CSWKYWFGEC	[137]
Peptide-conjugated NPs	P3-PLGA-PEG-MAL NP	[141]
Peptide-conjugated NPs	iRGD(CRGDK/RGPD/EC, a endothelial targeting peptide and specifically bind to integrinαvβ3/β5 receptor)-PLGA-PEG-MAL NP	[141]
PTNP	a PEGylated form of Pep-PEG5 kDa-NP. This information was modified from Ref [142]	[142]
tPep-PEG-LPs	P3 (GKGGRAKDGGC-NH2, purity: 93.6%, theoretical MW: 1004.15) -PEG-LPs. This information was modified from Ref [143]	[143]
Wave Life Sciences' extrahepatic delivery	GalNAc conjugates with PN	[144]

ATS-9R, adipocyte-targeting sequence and 9-arginine; APMAP, adipocyte plasma membrane-associated protein; C16, 2′-O-hexadecyl; GalNAc, N-acetylgalactosamine; LPs, liposomes, NLCs, nanostructured lipid carriers; NP, nanoparticle; PEG-LPs, maleimide-PEG2000-DSPE (PEG)-modified LPs; PLGA-PEG, poly (lactide-coglycolide)-*b*-poly (ethylene glycol); PN, phosphoryl guanidine; tPep, targeting peptide; TRiM™, targeted RNAi molecule, tPep-PEG-LPs: peptide ligand-mediated nanocarrier

### Specific EAT/PVAT targets

#### ADIPOQ targeting delivery strategies

According to genes from the NCBI and GeneCards, ADIPOQ is expressed exclusively in AT and encodes secretory ADPN. ADPN has multifarious beneficial effects on the body, such as improving AT vascularity and glucose/lipid metabolism, promoting insulin sensitivity and ceramide degradation, and suppressing oxidative stress and inflammation [145, 146]. ADPN has become a therapeutic target for multiple diseases, such as Alzheimer's disease, cardiovascular diseases, certain cancers, myocardial ischemia/reperfusion (I/R) injury, nonalcoholic fatty liver disease (NAFLD), neurodegenerative diseases, sarcopenia, and T2DM [147, 148]. Many agents have been developed in preclinical and clinical trials to target ADPN (Table 4), including ADIPONIN™ Solution 200-W [149–151], ADPN399 [152], ADPN400 (also named Chex-DSer-8) [152, 153], ALY688 (also named Adiponectin peptide analog-Allysta Pharmaceuticals, ADPN355, ALY688, ALY732, ALY-688-SR) [154–156], Antiagin II™ [157], Brightin™ [157], Clarin™ [157], CTRP1 (also named ADPN-like protein, C1QTNF1, CTRP1, zsig37) [158–160], Hydrin™ [157], SL100 [161–164], and PX811013 (Glycosylated ADPN) [165–168]. However, only ALY688 entered clinical trials. ADIPONIN™, Antiagin II™, Brightin™, Clarin™, and Hydrin™ are only used as healthcare products.

**Table 4:** The agents in clinical trials targeting ADPN and PHB1

Name	Targets	Administration	Structure/introduction	Status	Disease	Developer	Refs
ADIPONIN™ Solution 200-W	ADPN (agonist), AdipoR1/2 (agonist), filaggrin (agonist)	Topical	Water (and) 1,2-Hexanediol (and) Caprylyl (Butylene) Glycol (and) Acetyl Tripeptide-54 Amide. A tripeptide derived from adiponectin hormone	Approved	Anti-wrinkle, regeneration- and anti-aging agent	Supadelixir Co., Ltd	[149–151, 157]
ADPN399	ADPN (agonist), AdipoR1/2 (agonist)	IP, SC	ADPN355 linear branched dimer (H-DAsn-Ile-Pro-Nva-Leu-Tyr-DSer-Phe-Ala-DSer-His-Pro)2-Dab-NH2	Preclinical trials	Xerophthalmia, Hepatic fibrosis, NASH	Temple University	WO2019036223 (A1), [152]
ADPN400	ADPN (antagonist), AdipoR1/2 (antagonist)	IP, SC	H-Chex-Gly-Leu-Tyr-DSer-Phe-Ala-DSer-NH2	Preclinical trials	Breast cancer, chronic myeloid leukemia;	Temple University	[152, 153]
ALY688	ADPN (agonist), AdipoR1/2 (agonist)	An ophthalmic formulation of ALY688	ADPN-short peptide (H-DAsn-Ile-Pro-Nva-Leu-Tyr-DSer-Phe-Ala-DSer-NH2)	Phase 1/2a (Completed on 22 June 2023)	DED	Allysta Pharmaceutical	NCT04201574, [154]
ALY688				Phase 2b/3 (Completed on 16 March 2023)	DED	Allysta Pharmaceutical	NCT04899518, WO2019036223 (A1)
ALY688		SC (a sustained release systemic formulation of ALY688)		Phase 1 (Terminated due to recruitment is not possible due to COVID-19 pandemic restrictions on 30 August 2021)	Generally healthy overweight or Obese	Allysta Pharmaceutical	NCT04855565
ALY688		SC		Phase 1 (No report on 28 May 2024)	NASH	Allysta Pharmaceutical	[156]
Antiagin II™	ADPN, EGF, FGF-1, NFκB	Topical	Butylene Glycol (and) Palmitoyl sh-Tripeptide-3 Amide (and) Palmitoyl sh Tripeptide-1 Amide (and) Acetyl Tripeptide-54 Amide (and) Acetyl Tripeptide-74 Amide	Approved	Skin regeneration, Anti-aging	Supadelixir Co., Ltd	[157]
Brightin™	ADPN, MITF, Sfrp5	Topical	Butylene Glycol (and) Palmitoyl Tripeptide-71 Amide (and) Palmitoyl Tripeptide-53 Amide (and) Acetyl Tripeptide-54 Amide	Approved	Anti-pigmentation, Brightening	Supadelixir Co., Ltd	[157]
Clarin™	ADPN, FGF-1, SFRP5	Topical	Butylene Glycol (and) Palmitoyl sh-Tripeptide-1 Amide (and) Palmitoyl Tripeptide-54 Amide (and) Palmitoyl Tripeptide-53 Amide	Approved	Anti-aging, Brightening, Skin moisturizing	Supadelixir Co., Ltd	[157]
CTRP1	PAF (inhibitor)	IV	An ADPN paralogs	Preclinical trials (Discontinued on 14 September 2010)	Thrombosis	Zebra Biologics Inc. and Seattle Life Sciences	WO-09904000, [158, 160]
Hydrin™	ADPN, SFR5	Topical	Butylene Glycol (and) Acetyl Tripeptide-54 Amide (and) Palmitoyl Tripeptide-53 Amide	Approved	Skin barrier enhancing, Skin regeneration, Skin moisturizing	Supadelixir Co., Ltd	[157]
SL100	ADPN (agonist), AdipoR1/2 (agonist)	Topical SL100 (1%)	A small synthetic molecule agonist and structure unknown	Preclinical trials	DED	Senelix Co Ltd	[161–164]
		Unknown			NASH		
PX811013	FAS (inhibitor), insulin (Stimulant)TNFα (Inhibitor),	Unknown	A highly active novel isoform (glycoprotein) of full-length ADPN	Preclinical trials	Alcoholic hepatitis, autoinflammatory disorders, NASH, T2DM	Protemix Corp Ltd	[165–167]
PX811013				Discontinued on 30 June 2010	Alcoholic hepatitis, NASH		[168]
Adipotide™	PHB1(modulators), ADPN (agonist)	SC	A proapoptotic peptide (CKGGRAKDC-GG-D(KLAKLAK)2, specifically bind to PHB1)	Phase 1 (Discontinued on 18 January 2019)	Obesity	Arrowhead Pharmaceuticals	NCT01262664, U.S. Patent Nos. 7452964, 7,951,362, 8,252,764, 8,846,859, 8,067,377, [188, 189, 195]
KLA-PTNP	PHB1(modulators), ADPN (agonist)	IV	(This information was modified from Ref [190])	Preclinical trials	Obesity, metabolic syndrome	Hokkaido University	[190]

Types and groups were obtained from Adisinsight, Bing, Chinadrugtrials, ClinicalTrials, Glgoo, PharnexCloud, PubChem Compound, Pubmed, and Zhihuiya. AdipoR1, adiponectin receptor-1; CTRP1, C1q/tumor necrosis factor-related protein 1; COVID-19, coronavirus disease 2019; DED, dry eye disease; EAE, experimental autoimmune encephalomyelitis; FAS, fatty acid synthetase; I/R, ischemia/reperfusion; IP, intraperitoneal; NASH, nonalcoholic steatohepatitis; PAF, platelet activating factor; RA, rheumatoid arthritis; SC, subcutaneous; T2DM, type 2 diabetes mellitus

**Fig. 5:**
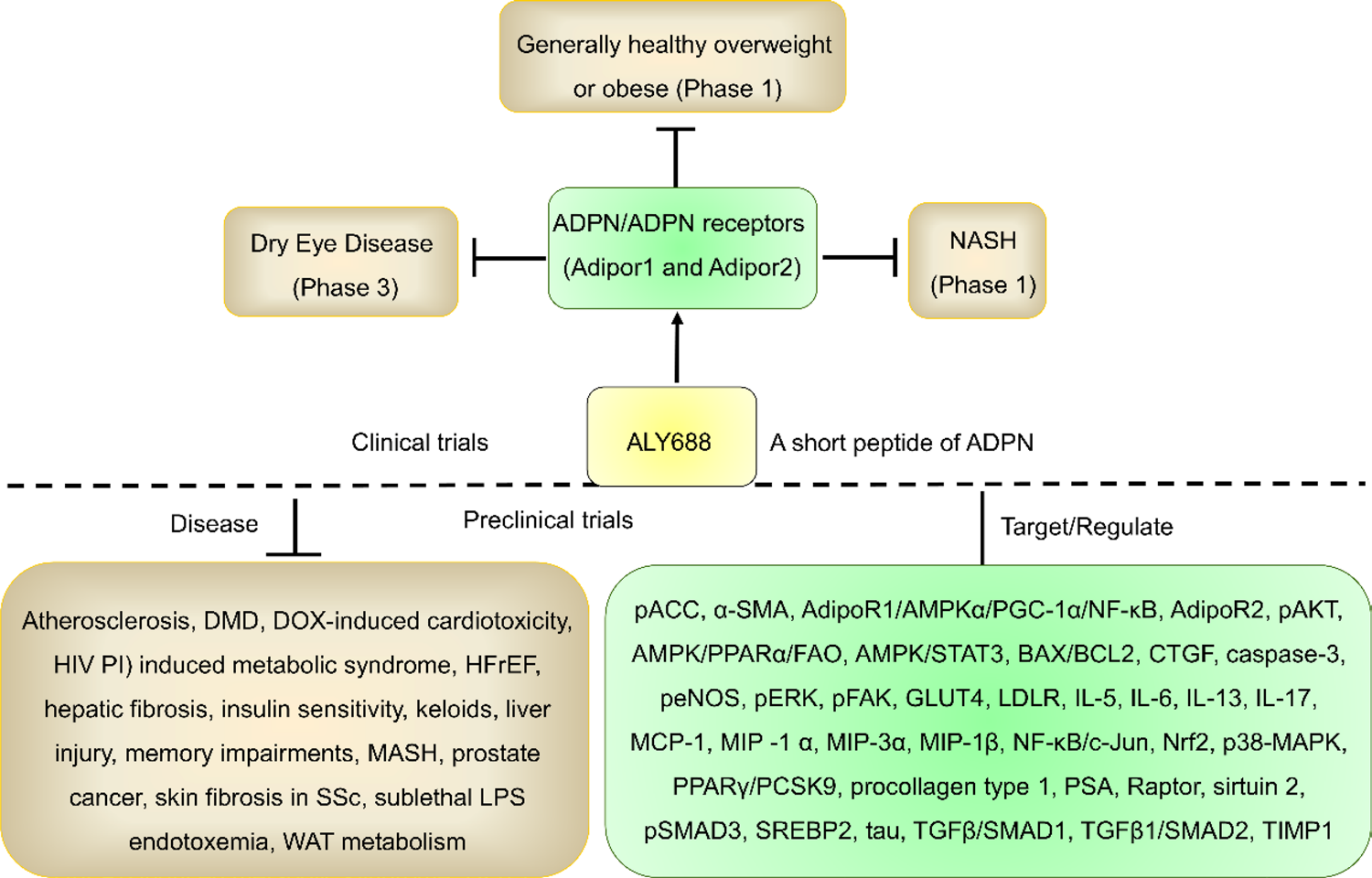
Development progress and targets of ALY688. ALY688 was investigated for the treatment of dry eye disease (Phase 3), generally healthy overweight or obese individuals (Phase 1), and NASH (Phase 2). According to preclinical trials, ALY688 has multiple targets and good efficacy and safety profiles against a variety of diseases

ALY688, a short peptide of ADPN, was developed by Allysta Pharmaceutical for the treatment of dry eye disease (DED), generally healthy overweight or obese individuals, and nonalcoholic steatohepatitis (NASH). According to preclinical studies, ALY688 has favorable effects and safety profiles for a variety of diseases, including atherosclerosis [169], duchenne muscular dystrophy (DMD) [170], doxorubicin (DOX)-induced cardiotoxicity [171], HIV protease inhibitor (PI)-induced metabolic syndrome [172], HFrEF [173], hepatic fibrosis [174, 175], insulin sensitivity [176], keloids [177], liver injury [178], memory impairments [179], metabolic dysfunction-associated steatohepatitis (MASH) [180], prostate cancer [181], skin fibrosis in systemic sclerosis (SSc) [182], sublethal LPS endotoxemia [183], and white AT (WAT) metabolism [184]. However, ALY688 not only regulated ADPN but also other genes (Fig. 5), including acetyl-CoA carboxylase (ACC) phosphorylation, α-smooth muscle actin (α-SMA), AdipoR1/AMPKα/PGC-1α/NF-κB, AdipoR2, AKT phosphorylation, AMPK/PPARα/FAO, AMPK/STAT3, BCL2-associated X protein/B-cell lymphoma 2 (BAX/BCL2), connective tissue growth factor (CTGF), caspase-3, eNOS phosphorylation, ERK phosphorylation, focal adhesion kinase (FAK) phosphorylation, GLUT4, LDLR, IL-5, IL-6, IL-13, IL-17, MCP-1, MIP-1 α, MIP-3α, MIP-1β, NF-κB/c-Jun, Nrf2, p38-MAPK phosphorylation, PPARγ/PCSK9, procollagen type 1, prostate-specific antigen (PSA), Raptor, sirtuin 2, SMAD3 phosphorylation, SREBP2, tau, TGFβ/SMAD1, TGFβ1/SMAD2, and tissue inhibitor of metalloproteinase I (TIMP1) [169–185]. Therefore, ALY688 had serious off-target effects.

#### PHB1 targeting delivery strategies

PHB1 is an AT vascular biomarker and plays a key role in aging and tumor suppression [186, 187]. Many agents have been developed in preclinical and clinical trials by targeting PHB1 (Table 4), including adipotide™ (also named KLA, Prohibitin targeting peptide 1 and Prohibitin-TP01) [188, 189] and KLA-PTNP [190]. Preclinical studies have shown that adipotide™ has good efficacy and safety for treating obesity [191, 192] and T2DM [193, 194]. KLA-PTNP, a PTNP conjugated to adipotide™, enhances the delivery of adipotide™ to the AT [190]. Adipotide™ and KLA-PTNP also increase ADPN levels [190, 192]. However, further development of adipotide™ has been limited for unknown reasons [195]. Notably, adipotide™ was developed by Arrowhead Pharmaceuticals [188, 189]. However, according to AdisInsight’s website, adipotide™ was developed by Ablaris Therapeutics [195]. In fact, Ablaris Therapeutics is a majority-owned subsidiary of Arrowhead Pharmaceuticals [196, 197]. However, AdisInsight did not disclose the relationship between Ablaris Therapeutics and Arrowhead Pharmaceuticals [195]. We are calling for changes to the information to ensure its timeliness.

#### RNA activation technology

RNA activation technology is a very scarce platform technology in the pharmaceutical field and has broad application prospects in the development of therapeutic drugs for cancer, genetic diseases, chronic diseases, metabolic diseases, and cardiovascular and cerebrovascular diseases. Self-amplifying RNA (saRNA), an RNA activation technology, uses double-stranded RNA that targets gene promoter regions to activate gene expression to restore the levels of therapeutic proteins. Today, many saRNAs were developed in preclinical and clinical trials (Table 5), including dsVHL-821 [198], MTL-CEBPA (also named MTL-001 and MTL-501) [199–203], MTL-011, Pidacmeran (also named BNT-162 program, BNT-162c2, PF-07302048 program) [204–206], Lipid nanoparticle (LNP)-nCoVsaRNA (saRNA vaccine, COVAC1) [207–210], RAG-01 [211], RAG-03 [211], RAG-05 [211], RAG-06 [211], RAG-12 [211], RAG-18 [211], RAG-1C [211], RAG-20 [211], RAG1-40-31L [212], Undisclosed [213–215], HbF (MiNA Therapeutics) [213], MTL-STING [158, 213, 216], LNP-encapsulated saRNA COVID-19 vaccine (VLPCOV-01) [217], Z004 [218], Z006 [218], Z007 [218], Z008 [218], and ZIP1642 [219]. Considering saRNA, direct overexpression of ADIPOQ and PHB1 may be beneficial for disease treatment.

**Table 5:** SaRNAs in preclinical and clinical trials

Name	Targets	Administration	Introduction	Status	Disease	Developer	Refs
dsVHL-821	VHL	Unknown	(This information was modified from Ref [198])	Preclinical trials	RCC	Ractigen Therapeutics	[198]
MTL-CEBPA	CEBPA (C/EBPα)	IV	MTL-CEBPA consists of a double stranded RNA formulated into a SMARTICLES® liposomal nanoparticle and is designed to activate the CEBPA gene (This information was modified from Ref [199])	Phase 1a/1b (Completed on 18 August 2021)	Advanced Solid Tumors	Mina Alpha Limited	NCT02716012, [202, 203]
MTL-CEBPA	CEBPA	IV		Phase 1 (Recruiting on 28 October 2021)	Advanced HCC	Mina Alpha Limited	NCT05097911 (Plus Atezolizumab or Bevacizumab)
MTL-CEBPA	CEBPA	IV		Phase 1a/1b (Active, not recruiting on 5 June 2023)	Advanced Solid Tumors	Mina Alpha Limited	NCT04105335 (Plus PD-1 Inhibitor pembrolizumab)
MTL-CEBPA	CEBPA	IV		Phase 2 (Active, not recruiting on 27 November 2023)	TKI naive with Previously Treated Advanced HCC, HBV, HCV	Mina Alpha Limited	NCT04710641 (Plus Sorafenib)
MTL-011	CEBPA	IV		Preclinical trials	T2DM	MiNA Therapeutics Limited	WO2015075557 (A2 and A3)
Pidacmeran	SARS-CoV-2 Spike glycoprotein	IM	A vaccine based on self-amplifying mRNA33	Phase 1/2 (Unknown status)	COVID-19	BioNTech SE	[206]
LNP-nCoVsaRNA	SARS-CoV-2 Spike glycoprotein	IM	A saRNA SARS-CoV-2 vaccine candidate	Phase 1 (Completed on 4 October 2022)	COVID-19	MRC/UVRI and LSHTM Uganda Research Unit	[207]
LNP-nCoVsaRNA	SARS-CoV-2 spike protein	IM		Phase 2a (Completed on 13 January 2023)	COVID-19	MRC/UVRI and LSHTM Uganda Research Unit	[208]
LNP-nCoVsaRNA				Phase 1 (Completed on 14 January 2022)	COVID-19	MRC/UVRI and LSHTM Uganda Research Unit	[210]
RAG-01	P21	Bladder instillation	Urothelial cells targeting by Ractigen’s proprietary LiCO™ delivery technology promote the expression of the anticancer gene p21, which is low or no expression in bladder cancer cells	Phase 1 (Recruiting on 28 May 2024)	NMIBC unresponsive to BCG therapy	Ractigen Therapeutics	NCT06351904, [211]
RAG-03	THPO	Undisclosed	Liver targeting promotes the expression of THPO, which is a hematopoietic growth factor and stimulates platelet production	Preclinical trials (Lead development)	Persistent thrombocytopenia	Ractigen Therapeutics	[211]
RAG-05	HMBS	Undisclosed	Liver targeting promotes the expression of HMBS, a metabolic enzyme and lacking that will induce AIP	Preclinical trials (Lead development)	AIP	Ractigen Therapeutics	[211]
RAG-06	SMN2	Undisclosed	Increases the level of SMN (motor neuron survival), which is necessary for the normal function of motor neurons	Preclinical trials (Lead development)	SMA	Ractigen Therapeutics	[211]
RAG-12	SERPING1	Undisclosed	Liver targeting promotes the expression of SERPING1, a code gene for C1-Inh, lacking that will induce HAE	Preclinical trials (Lead development)	HAE	Ractigen Therapeutics	[211]
RAG-18	UTRN	Undisclosed	Muscle tissue targeting by Ractigen’s proprietary LiCO™ technology to increase muscle UTRN expression	IND-enabling	DMD	Ractigen Therapeutics	[211]
RAG-1C	Cell cycle inhibitory gene	Vitreous injection	An agent that inhibits the proliferation of RPE cells by activating the cyclin suppressor gene	IND-enabling	PVR	Ractigen Therapeutics	[211]
RAG-20	FVII	Undisclosed	Liver targeting by Ractigen's proprietary GLORY™ technology promotes liver FVII production	Preclinical trials (Lead development)	FVII deficiency/Hemophilia with inhibitors	Ractigen Therapeutics	[211]
RAG1-40-31L	p21	Intravesical administration	A lipid conjugate targeting p21	Preclinical trials	NMIBC	Ractigen Therapeutics	[212]
Undisclosed	Undisclosed	Undisclosed	Undisclosed	Discovery	Undisclosed	MiNA Therapeutics	[213]
Undisclosed	Up to 5 targets	Undisclosed	Undisclosed	Discovery	immuno-oncology, genetic diseases	MiNA Therapeutics and Eli Lilly	[213–215]
Liposome-HbF	HBG	IV	Promote HbF (a redundant form of hemoglobin) production by increasing HBG transcription	Preclinical trials (Discovery)	SCD and Beta Thalassemia	MiNA Therapeutics	[213]
MTL-STING	STING1	Undisclosed	Myeloid cells targeting by NOV340 liposomes (A clinically proven myeloid cell delivery vector) promote STING expression (4 to 11-fold) by enhancing transcriptional activation across the whole STING (Inhibition of immune escape and activation of cGAS-cGAMP-STING pathway) locus	IND-enabling	Advanced Solid Tumors	MiNA Therapeutics	[158, 213]
VLPCOV-01	SARS-CoV-2 spike protein	Booster injections	An LNP encapsulated saRNA vaccine that expresses the membrane-anchored S-RBD of SARS-CoV-2	Phase 1 (Completed on 15 August 2023)	COVID-19	VLP Therapeutics Japan, Inc	[217]
Z004	Undisclosed	Undisclosed	Undisclosed	Preclinical trials	Cystic Fibrosis	Ziphius Vaccines	[218]
Z006	Undisclosed	Undisclosed	LNP-based delivery to protect RNA molecules from RNase-mediated degradation	Discovery	Undisclosed	Ziphius Vaccines	[218]
Z007	Undisclosed	Undisclosed	Undisclosed	Preclinical trials	*Chlamydia trachomatis*	Ziphius Vaccines	[218]
Z008	Undisclosed	Undisclosed	Undisclosed	Preclinical trials	Oncology	Ziphius Vaccines	[218]
ZIP1642	SARS-CoV-2 S-RBD, N antigen	IM	an LNP-formulated dual-antigen saRNA vaccine that encodes SARS-CoV-2 S-RBD and the N antigen	Preclinical trials	COVID-19	Ziphius Vaccines	[219]

Types and groups were obtained from Adisinsight, Bing, Chinadrugtrials, ClinicalTrials, Glgoo, PharnexCloud, PubChem Compound, Pubmed, and Zhihuiya. AIP, acute intermittent porphyria; BCG, Bacillus Calmette Guérin; C1-Inh, C1-esterase inhibitors; COVID-19, coronavirus disease 2019; DMD, Duchenne muscular dystrophy; HAE, hereditary angioedema; HbF, fetal hemoglobin; HBV, hepatitis B virus; HCC, hepatocellular carcinoma; IM, intramuscular; IV, intravenous; IP, intraperitoneal; LNP, lipid nanoparticle; NMIBC, non-muscle invasive bladder cancer; S-RBD, receptor-binding domain of spike protein; RCC, renal cell carcinoma; PVR, proliferative vitreoretinopathy; SCD, sickle cell disease; SMA, spinal muscular atrophy; T2DM, type 2 diabetes mellitus; TKI, tyrosine kinase inhibitor

## Future directions and challenges

Vasogenic inflammatory molecules induce perivascular lipolysis, resulting in the transformation of small fat cells that are low in fat around the inflamed arteries into large fat cells that are high in fat. Therefore, EAT and PVAT can be used as a sensor to detect the danger of cardiovascular disease in the blood vessel wall, and the detection of EAT and PVAT can reflect the state of vascular inflammation. Previous studies have shown that imaging detection of EAT and PVAT (such as fat attenuation index (FAI) detection and CT detection) is superior to that of vascular circulation inflammation markers, with more high-risk plaque characteristics, and is more conducive to anti-inflammatory therapy and the prognosis of major cardiovascular events. The EAT and PVAT increase with age and may serve as therapeutic targets for the modulation of fat in patients with diabetes and obesity-related cardiovascular risk. CT is the gold standard for detecting PVAT and EAT. However, the cost of CT detection is greater than that of circulating marker detection. Determining how to accurately detect PVAT and EAT and reduce costs is a research direction that needs important breakthroughs in the future. Inhibition of EAT/PVAT thickness and inflammation is one of the pharmacological mechanisms by which many drugs inhibit the development of atherosclerosis, including DPP4 inhibitors (such as alogliptin, saxagliptin, sitagliptin, and teneligliptin, GLP-1R agonists (such ase dulaglutide, exenatide, liraglutide, and semaglutide), and SGLT-2i (such as canagliflozin, dapagliflozin, empagliflozin, ipragliflozin, and luseogliflozin). Pharmacological in combination with non-pharmacological, such as regular physical exercise and plant-based diet, could be more effective in reducing cardiovascular risk than drugs alone. Notably, only DPP4 was more highly expressed in EAT and PVAT, suggesting that DDP4 is a promising target for targeting EAT and PVAT formulations. However, further research is needed to determine how to reduce off-target effects or enhance EAT and PVAT delivery. Many AT delivery materials were developed. However, more studies are needed to confirm the feasibility of the material for clinical use. PHB1 is an AT vascular biomarker, and ADIPOQ is only expressed in AT and encodes ADPN. Many ADPN and PHB1 agents have been developed in preclinical and clinical trials. However, these agents have serious off-target effects. RNA activation technology, such as saRNA, is a good technique for drug development to reduce off-target effects. Several saRNA agents are already under clinical investigation. Taken together, many strategies may be more conducive to the development of targeting EAT and PVAT agents, including AT delivery strategies, ADIPOQ/PHB1-targeted therapies, and saRNA technology. However, we did not discuss these strategies in detail. This is the biggest weakness. With the deepening of research, the advancement of technology, and the cooperation of scientific research, we believe that there will be targeted EAT and PVAT preparations, such as ADIPOQ and PHB1 saRNA.

## Data Availability

No datasets were generated or analysed during the current study.

## Abbreviations

ACC: Acetyl-CoA carboxylase
ADPN: Adiponectin
AGEs: Advanced glycation end products
AGT: Angiotensinogen
ASCVD: Atherosclerotic cardiovascular disease
α-SMA: α-Smooth muscle actin
AT: Adipose tissue
BAX/BCL2: BCL2-associated X protein/B-cell lymphoma 2
C16: Centyrin ligands, 2′-O-hexadecyl
CAD: Coronary artery disease
CHD: Coronary heart disease
CTGF: Connective tissue growth factor
DED: Dry eye disease
DMD: Duchenne muscular dystrophy
DOX: Doxorubicin
DPP4: Dipeptidyl peptidase 4
EAT: Epicardial adipose tissue
ECs: Endothelial cells
eNOS: Endothelial NO synthase
FAK: Focal adhesion kinase
GalNAc: N-acetylgalactosamine
GLP-1: Glucagon-like peptide-1
GLUT4: Glucose transporter type 4
HAT: Heart adipose tissue
HFrEF: Heart failure with reduced ejection fraction
I/R: Ischemia/reperfusion
IMAT: Intra-myocardial adipose tissue
LDL-c: Low-density lipoprotein cholesterol
LNP: Lipid nanoparticle
MASH: Metabolic dysfunction-associated steatohepatitis
MCP-1: Monocyte chemoattractant protein-1
MI: Myocardial infarction
MMe: Metabolically activated macrophages
Mox: Oxidized macrophages
NAFLD: Nonalcoholic fatty liver disease
NASH: Nonalcoholic steatohepatitis
NCBI: National Center for Biotechnology Information
NGF: Nerve growth factor
NO: Nitric oxide
PAI-1: Plasminogen activator inhibitor-1
PHB1: Prohibitin 1
PI: Protease inhibitor
PN: Phosphoryl guanidine
PSA: Prostate-specific antigen
PVAT: Perivascular adipose tissue
saRNA: Self-amplifying RNA
SGLT-2: Sodium glucose cotransporter 2
SGLT-2i: SGLT-2 inhibitors
T2DM: Type 2 diabetes mellitus
Tc1: T cytotoxic 1
TIMP1: Tissue inhibitor of metalloproteinase I
WAT: White AT
